# Recurrent Erythema Multiforme Major Following COVID-19 Infection

**DOI:** 10.7759/cureus.42646

**Published:** 2023-07-29

**Authors:** Kayla Rykiel, Julian Melchor, Ian Motie, Kevin Mulles, Vida Farhangi

**Affiliations:** 1 Medicine, Florida State University College of Medicine, Tallahassee, USA; 2 Internal Medicine, Florida State University School of Medicine, Sarasota Memorial Hospital, Sarasota, USA

**Keywords:** stevens-johnson syndrome, herpes simplex, recurrent erythema multiforme, sars-cov-2, coronavirus disease, covid-19, erythema multiforme-like lesions, erythema multiforme major

## Abstract

Erythema multiforme (EM) is a rare and potentially serious skin condition that can present as a myriad of mucocutaneous lesions. EM can be commonly confused with other cutaneous etiologies, leading to misdiagnosis and delay in proper treatment. This paper describes a case of recurrent erythema multiforme following COVID-19 infection in a 34-year-old male with no prior medical history. The patient had an extensive rash of the oral and genital mucosal areas, diffuse cutaneous involvement, and an extended length of recovery. This unexpected association of EM and COVID-19 provides additional insight into the limited research available regarding the correlation between these two pathologies.

## Introduction

Erythema multiforme (EM) is a potentially serious immune-mediated, hypersensitivity reaction of the skin, often recognized by its pathognomonic targetoid lesions. It is most commonly triggered by infections such as herpes simplex virus (HSV), *Mycoplasma pneumoniae*, Epstein-Barr virus (EBV), and HIV and less commonly by reaction to medications or from immunosuppression [1-4]. Two different staging categories of EM exist, EM minor and EM major. EM minor presents with typical lesions that rarely involve the mucosa [5], whereas EM major presents with skin lesions that are more severe and often involve at least two different mucosal sites [5]. While some reports of self-limited EM are associated with COVID-19, little is known about recurrent EM major outbreaks following COVID-19 infection. In this case, we discuss the presentation of a persistent rash following COVID-19 and our approach to diagnosis and treatment.

## Case presentation

A 34-year-old male with no reported past medical history presented to the emergency department with a diffuse rash accompanied by subjective fever, nausea, vomiting, diarrhea, and oral thrush. Over the preceding 48 hours, the rash progressively worsened and spanned his thighs, groin, forearms, wrists, ankles, palms, and soles. He described large erythematous, plaque-like lesions with distant lesions having a targetoid appearance. He noted that this was his sixth episode of the rash, although previous episodes had not been this extensive. He first noticed the rash one month after being diagnosed with COVID-19 in August 2020, two years before his current presentation. He subsequently experienced five episodes of the rash during the next seven months. Prodromal symptoms consisted of subjective fevers followed by circular lesions on his wrists, groin, and lower extremities that took up to six weeks to resolve. In February of 2022, he visited urgent care and was trialed on oral doxycycline and oral corticosteroids for the empiric treatment of the rash with minimal relief of symptoms. No cultures were taken at the time, and no specific COVID-19 treatments, aside from supportive care, were provided. He denied any other medications and was fully vaccinated against the COVID-19 virus on presentation.

**Figure 1:**
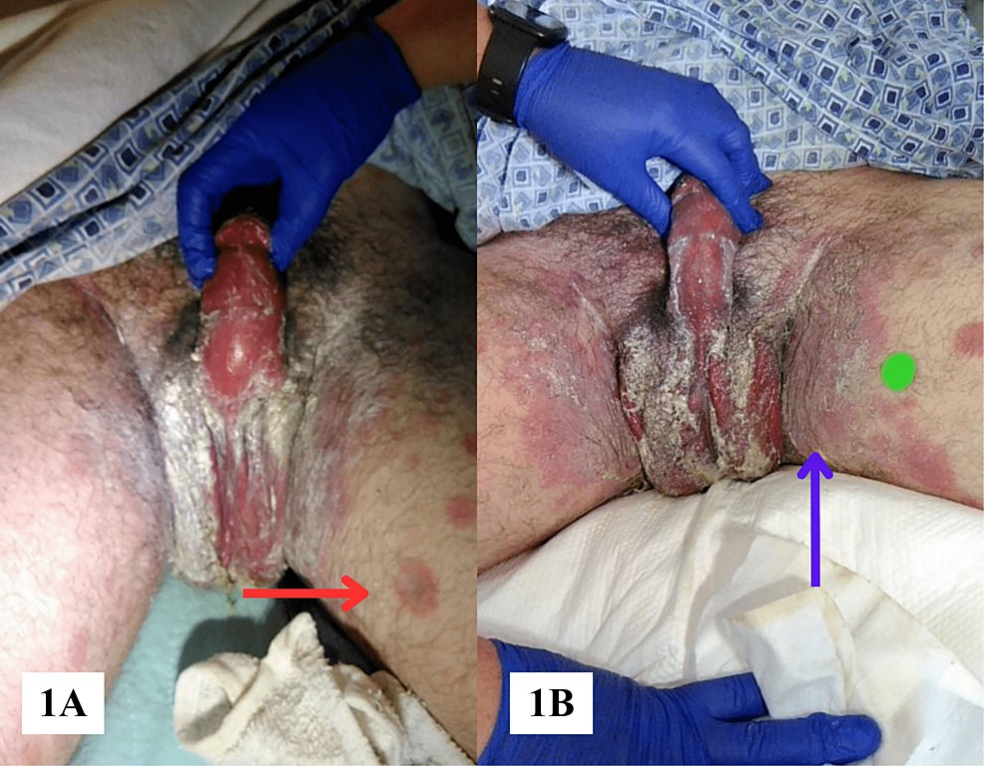
Erythema multiforme major shown as erythematous, dusky targetoid papules and plaques of the medial thighs (red arrow) coalescing into large eroded plaques in the bilateral groin (blue arrow).

**Figure 2:**
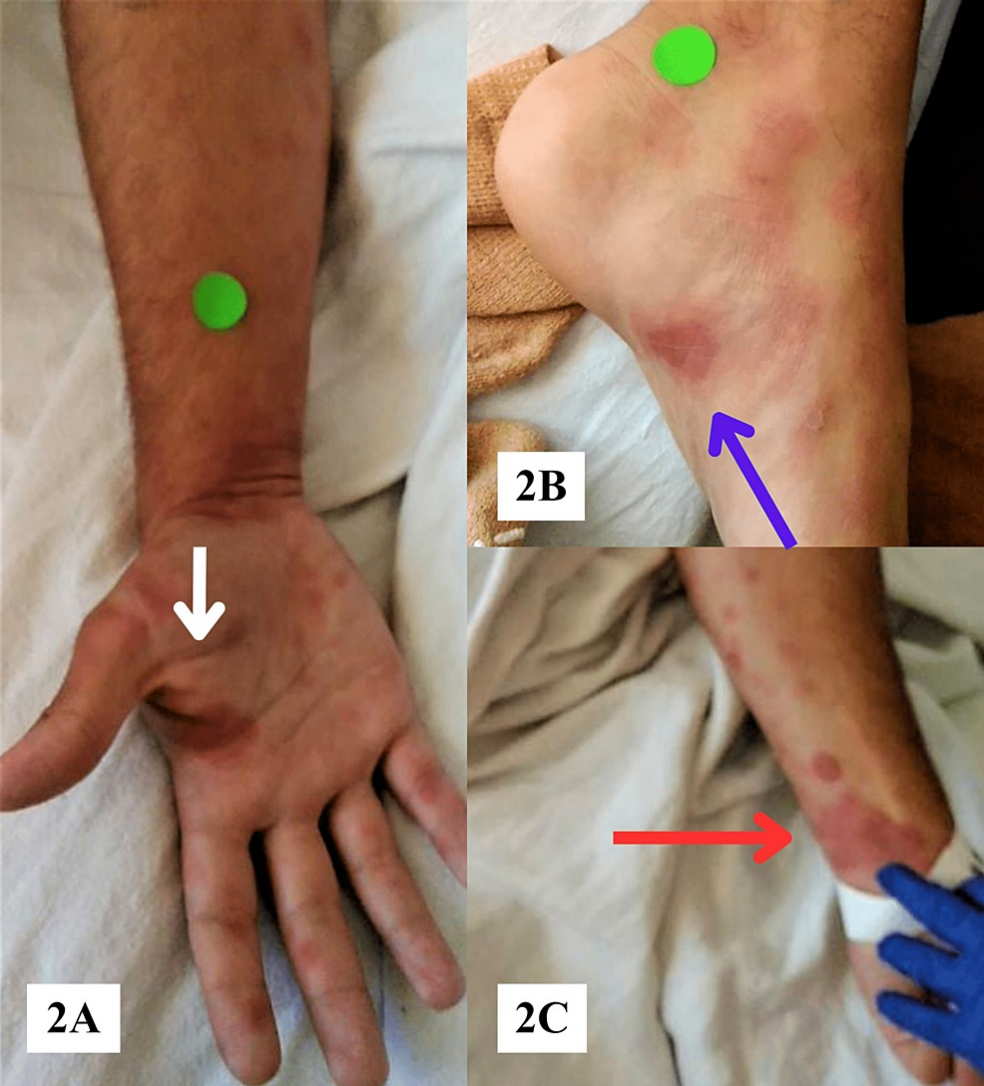
Erythema multiforme lesion manifestation on the extremities. (A) Palmar surface of the right hand and wrist (white arrow). (B) Typical target lesion seen on the medial arch of the left foot (blue arrow). (C) Lesions on the volar aspect of the right wrist (red arrow)

On evaluation in August 2022, the patient's vital signs were within normal limits. His physical examination was significant for a severe rash in his groin with the inner thighs and scrotum appearing violaceous (Figure 1A, 1B). Additionally, he had a diffuse, erythematous rash symmetrically distributed with innumerable plaques measuring up to 3 cm on his wrists, forearms, elbows, thighs, and ankles (Figure 2A, 2B, 2C). Although his face was spared, his oral examination showed erythematous eroded plaques with vesicles. Complete blood count and metabolic panel were unremarkable (Table 1). An infectious and rheumatologic workup was positive only for HSV-1 IgG antibodies but negative for HIV, COVID-19, mononucleosis, syphilis, or autoimmune conditions. On further questioning, the patient denied any previous history of oral or genital lesions and never required antiviral treatment. He was started on nystatin oral suspension for the treatment of possible thrush and treated supportively with topical zinc oxide paste bandages.

**Table 1:** Patient's laboratory results upon admission to the hospital in August 2022 with reference ranges as indicated by the hospital system. AST, aspartate transaminase; ALT, alanine transaminase

Result	Patient's Results (Reference Range)
WBC	8.9×10^3^/uL (4.5-11.0×10^3^/uL)
RBC	5.38×10^6^/uL (4.50-5.90×10^6^/uL)
Hemoglobin	15.7 g/dL (14.0-17.5 g/dL)
Hematocrit	44.4% (40.0-52.0%)
Platelet	216×10^3^/uL (150-400×10^3^/uL)
Glucose	114 mg/dL (70-100 mg/dL)
Sodium	139 mmol/L (132-144 mmol/L)
Potassium	3.5 mmol/L (3.5-5.1 mmol/L)
Chloride	106 mmol/L (98-110 mmol/L)
Carbon Dioxide Serum	29 mmol/L (21-32 mmol/L)
Blood Urea Nitrogen	8 mg/dL (6-20 mg/dL)
Creatinine Serum	1.24 mg/dL (0.70-1.30 mg/dL)
Bilirubin Total	0.5 mg/dL (0.2-1.3 mg/dL)
Alkaline Phosphatase	102 U/L (26-162 U/L)
AST	20 U/L (15-37 U/L)
ALT	30 U/L (16-61 U/L)

**Figure 3:**
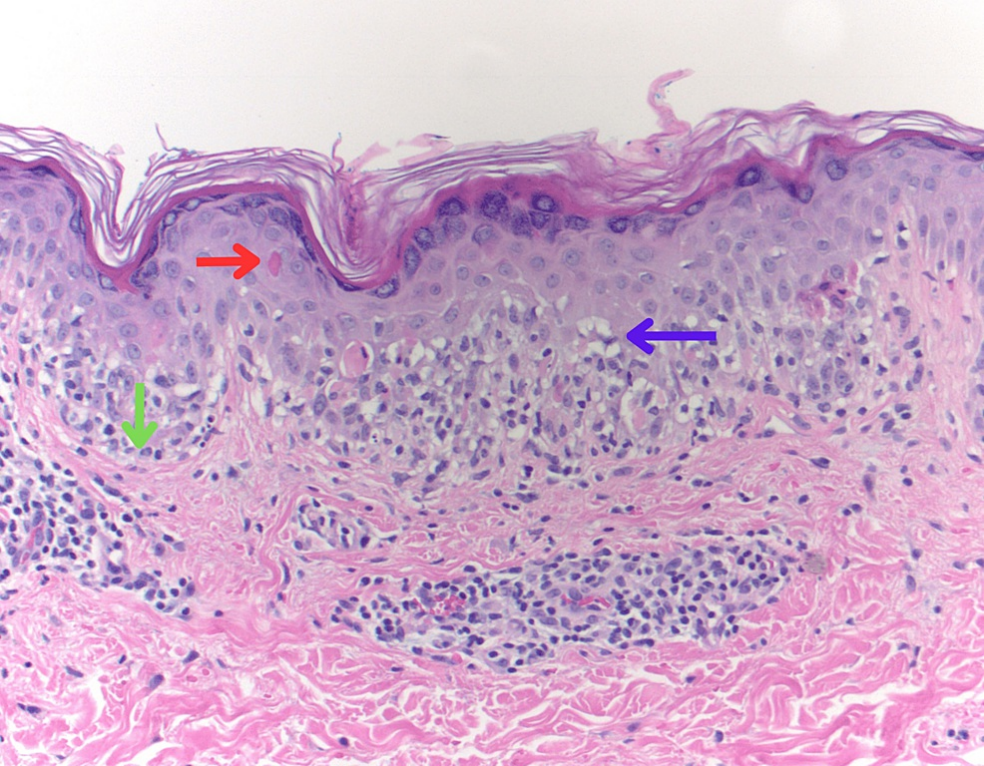
Erythema multiforme depicted in the biopsy specimen of the right thigh showing epidermal necrosis (red arrow), autoimmune destruction at dermal-epidermal junction (green arrow), and vacuolar interface changes (blue arrow) (hematoxylin & eosin stain at 200×).

He underwent a biopsy of his right thigh showing lichenoid dermatitis with keratinocyte necrosis with a differential that included EM but could not exclude early Stevens-Johnson syndrome (SJS) (Figure 3). He was evaluated by dermatology, who agreed that his diagnosis was more consistent with EM major based on his examination and biopsy. He was then started on valacyclovir 1000 mg daily for asymptomatic HSV-1 and oral antihistamines as needed for itching. He was safely discharged with close outpatient follow-up. At his one-month follow-up appointment, the patient was noted to have marked improvement in his rash and thrush and was recommended to continue taking his antivirals for an additional eight weeks. After six months, the patient reported complete resolution of the rash.

## Discussion

EM is commonly induced by infection and is associated with a targetoid rash but can present with atypical skin lesions [1,3]. A biopsy can assist when a diagnosis is unclear, and findings may support EM or exclude other pathologies. Once a diagnosis of EM is supported, determining the underlying etiology assists in identifying a treatment approach.

There are other reported cases of EM occurring after COVID-19 infection. According to recent literature reviews, COVID-19-induced EM seems to occur more frequently in patients younger than 30 years old or older than 55 years old [2]. This adds to the rarity of our patient's presentation since he did not fall under the juvenile type or older type of virus-related EM presentation [2]. Hydroxychloroquine infrequently was one drug that may have been associated with drug-induced EM in COVID-19 patients, but these patients mainly presented 3-10 days after drug initiation [2]. Some studies have also shown an association between EM and COVID-19 vaccination; however, this is unlikely in our case given that the patient presented with a year of recurrent EM rashes before being vaccinated. EM after COVID-19 vaccination in the majority of the patient population occurred three days after vaccination, which did not occur in this case [6].

Treatment for EM varies based on the etiology and severity. Minor disease can be treated supportively with topical corticosteroids or oral antihistamines, while major disease requires systemic steroids [3]. EM typically does not require hospitalization, except for severe cases, particularly when oral intake is limited due to mucosal lesions [1]. Recurrent disease is most likely from a viral etiology; thus, the investigation for viral etiology with subsequent treatment is warranted [1,3]. If no underlying cause can be identified, patients can be treated empirically with antivirals for up to six months [7]. Diseases nonresponsive to first-line therapy are trialed on azathioprine, mycophenolate mofetil, or dapsone [1,3]. Apremilast, a phosphodiesterase inhibitor, can also be considered for resistant disease [8].

The limitations of this case include the medications this patient had been treated with in the outpatient setting, including doxycycline. Doxycycline is a known culprit for SJS and EM reactions [9]. However, the patient had experienced multiple episodes of typical EM lesion outbreaks before eventually being prescribed doxycycline, making it highly unlikely that the medication played a role in his presentation.

## Conclusions

This case draws attention to the minimally reported relationship between EM and COVID-19. While some case reports have highlighted COVID-19 associated with a single episode of self-resolving EM, there is limited data documenting COVID-19 as an etiology of recurrent EM major. Although this patient was found to have subclinical HSV-1 infection of unknown chronicity, it is unlikely that this contributed to the patient's presentation. In most cases where HSV is the inciting factor, patients will present with typical HSV lesions prior to the onset of EM, which this patient never experienced.

Ultimately, determining an association between EM and COVID-19 could potentially change the course of treatment; however, more research is needed to establish a specific treatment protocol for this type of presentation.
